# Isopropanol Electro-Oxidation on PtCu Alloys for Aqueous Organic Redox Chemistry Toward Energy Storage

**DOI:** 10.3390/molecules30194027

**Published:** 2025-10-09

**Authors:** Jinyao Tang, Xiaochen Shen, Laura Newsom, Rongxuan Xie, Parsa Pishva, Yanlin Zhu, Bin Liu, Zhenmeng Peng

**Affiliations:** 1 Department of Chemical Engineering, University of South Carolina, Columbia, SC 29208, USA; jinyao@email.sc.edu (J.T.);; 2 Department of Chemical and Biomolecular Engineering, North Carolina State University, Raleigh, NC 27606, USA; 3 Tim Taylor Department of Chemical Engineering, Kansas State University, Manhattan, KS 66506, USA; binliu@ksu.edu

**Keywords:** isopropanol electro-oxidation, Pt-Cu alloy catalysts, activity, stability, redox flow batteries (RFBs)

## Abstract

Integration of renewable energy into modern power grids remains limited by intermittency and the need for reliable energy storage. Redox flow batteries (RFBs) are promising for large-scale energy storage, yet their widespread adoption is hindered by the high cost. In this study, we investigate isopropanol as a redox-active species with Pt-Cu alloy electrocatalysts for aqueous-organic RFBs. A series of Pt_x_Cu catalysts with varying Pt:Cu ratios were synthesized and studied for isopropanol electro-oxidation reaction (IPAOR) performance. Among them, PtCu demonstrated the best performance, achieving a low activation energy of 14.4 kJ/mol at 0.45 V vs. RHE and excellent stability at 1 M isopropanol (IPA) concentration. Kinetic analysis and in situ attenuated total reflectance-Fourier transform infrared (ATR-FTIR) spectroscopy revealed significantly reduced acetone accumulation on PtCu compared to pure Pt, indicating enhanced resistance to catalyst poisoning. Density functional theory (DFT) calculations further identified the first proton-coupled electron transfer (PCET) as the rate-determining step (RDS) with C-H bond scission as the preferred pathway on PtCu. A proof-of-concept PtCu-catalyzed H-cell demonstrated stable cycling over 200 cycles, validating the feasibility of IPA as a low-cost, regenerable redox couple. These findings highlight PtCu-catalyzed IPA/acetone(ACE) chemistry as a promising platform for next-generation aqueous-organic RFBs.

## 1. Introduction

The transition to renewable energy sources is a critical step toward achieving a sustainable and low-carbon energy future. While these sources are inherently clean and sustainable, their widespread integration is impeded by the intermittent nature of power generation and the high cost of infrastructure for long-distance electricity transmission [1,2]. To overcome these challenges, scalable and economically viable energy storage technologies are needed. Redox flow batteries (RFBs), with their modularity, decoupled energy and power capacity, and potential for grid-level deployment, are one of the most promising candidates for this role [3].

Among RFB technologies, organic-based RFBs have garnered significant attention due to the use of earth-abundant elements like carbon, hydrogen, oxygen, and nitrogen, offering a cost-effective and environmentally friendly alternative to conventional vanadium-based systems [4]. Redox-active organic compounds such as quinone and 2,2,6,6-Tetramethylpiperidine-1-oxyl (TEMPO) have been extensively studied [5,6], with many structural modifications studies to improve their applicability, including redox reversibility, electrolyte compatibility, and solubility [4,7,8,9,10]. However, these systems are often limited by high molar mass, complex synthesis routes, and limited theoretical capacities, which constrain their scalability and economic viability.

**Figure 1:**
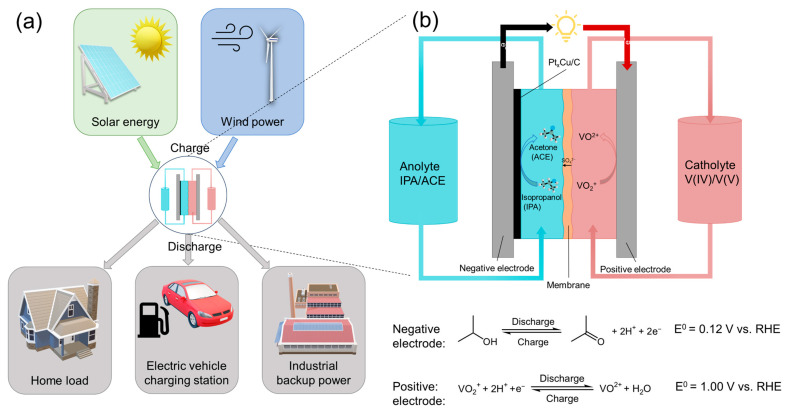
(**a**) Schematic drawing of potential applications of IPA/ACE flow battery; (**b**) Schematic drawing of flow battery with IPA/ACE as anolyte and V(IV)/V(V) as catholyte.

A promising yet underexplored class of redox-active molecules is small, low-cost secondary alcohols such as isopropanol (IPA), which forms a reversible redox couple with acetone (ACE). This IPA/ACE pair utilizes simple, safe, and commercially available chemicals, and offers significantly higher theoretical capacity compared to conventional organic molecules. The IPA/ACE redox pair operates via IPA electro-oxidation reaction (IPAOR) to ACE and its reverse electro-reduction, providing a unique opportunity to leverage alcohol-based chemistry for energy storage in RFBs (Figure 1) [11,12,13,14,15]. However, a critical barrier to realizing the potential of the IPA/ACE redox system lies in the IPAOR, which requires noble metal catalysts such as Pt. While Pt is considered the state-of-the-art catalyst for this reaction, it exhibits only moderate activity and suffers from poor long-term stability due to deactivation by strongly adsorbed intermediates [12,13,16,17]. This catalyst poisoning significantly impairs efficiency and cycle life, limiting practical application. Therefore, there is a pressing need to discover more active and durable electrocatalysts that not only enhance performance and resist deactivation but also reduce noble metal content, thereby enabling cost-effective and scalable IPA/ACE-based RFBs.

In this study, we report the discovery of Pt-Cu alloy nanoparticles as highly effective catalysts that significantly enhance both the activity and stability of IPAOR to ACE. By systematically tuning the Pt:Cu ratio, we identify PtCu as the optimal composition, exhibiting superior catalytic performance characterized by significantly enhanced IPAOR activity, low activation energy, and excellent activity retention during stability testing. Mechanistic studies combining electrochemical kinetics, in situ infrared spectroscopy, and density functional theory (DFT) calculations reveal that the enhanced activity originates from both kinetic and molecular-level improvements. Specifically, DFT results indicate that IPAOR on PtCu preferentially follows a C-H bond scission pathway with a lower energy barrier (0.73 eV) than the O-H scission route (1.01 eV). In situ attenuated total reflectance-Fourier transform infrared (ATR-FTIR) spectroscopy confirms that PtCu suppresses the formation of poisoning intermediates compared to pure Pt, thereby enhancing catalyst durability. Building on these insights, we demonstrate a proof-of-concept IPA/ACE-based H-cell using IPA/ACE as the redox couple and PtCu as the anode catalyst, paired with vanadium-based catholyte to provide a benchmark for evaluating IPA oxidation performance. The system exhibits stable charge–discharge operation over 200 cycles, four times the durability of Pt catalyst-based counterparts. This work not only identifies PtCu alloys as a robust and scalable electrocatalyst platform for IPAOR but also validates IPA/ACE as a promising high-capacity, low-cost redox pair for future aqueous-organic RFBs.

## 2. Results and Discussions

**Figure 2:**
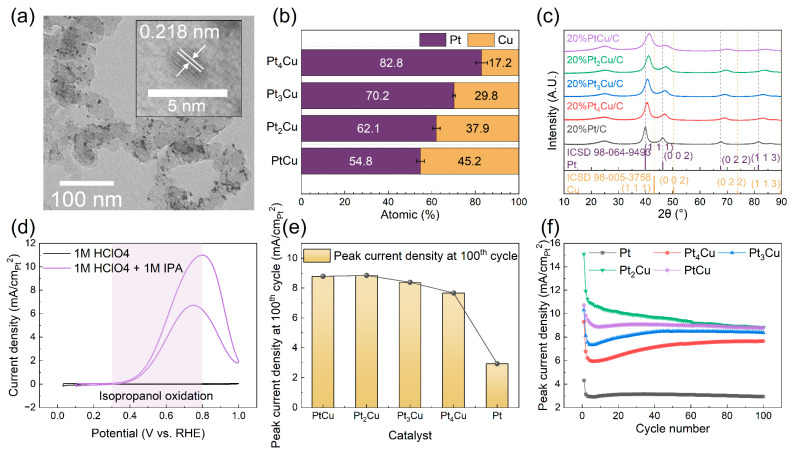
(**a**) TEM and HRTEM (insert) images of PtCu; (**b**) Elemental composition determined by EDS and (**c**) XRD patterns of Pt_x_Cu samples (x = 1, 2, 3, 4); (**d**) CV curves of PtCu in 1 M HClO_4_ and 1 M HClO_4_ + 1 M IPA, respectively, with potential window of 0–1.0 V vs. RHE; (**e**) Peak current densities of Pt_x_Cu samples (x = 1, 2, 3, 4) and Pt in 1 M HClO_4_ + 1 M IPA at 100th cycle; (**f**) Peak current densities of Pt_x_Cu samples (x = 1, 2, 3, 4) and Pt in 1 M HClO_4_ + 1 M IPA at 100th cycle.

The synthesized Pt_x_Cu (x = 1, 2, 3, 4) and pure Pt catalyst samples were initially characterized using transmission electron microscopy (TEM), energy-dispersive X-ray spectroscopy (EDS) and X-ray diffraction (XRD) to confirm successful synthesis and alloy formation. Figure 2a presents a representative TEM image of the PtCu sample, showing uniformly dispersed PtCu nanoparticles on the carbon support with an average particle size of approximately 2.4 nm (Figures S1 and S2). High-resolution TEM (HRTEM) analysis reveals distinct lattice fringes with a measured d-spacing of 2.218 Å, corresponding to the (111) plane. Compared to the d-spacing of pure Pt (0.222 nm, Figures S3 and S4), this lattice contraction confirms the successful formation of the PtCu alloy [18]. EDS analysis further verified that the Pt:Cu molar ratios matched the targeted compositions (Figure 2b). Thermogravimetric analysis (TGA) indicated a catalyst loading of approximately 20 wt.% Pt (Figures S5 and S6). XRD patterns of the Pt_x_Cu samples showed characteristic peaks corresponding to the face-centered cubic (*fcc*) structure (Figure 2c), with peak positions systematically shifting toward higher angles as the Cu content increased. This peak shift is consistent with the lattice shrinkage observed in HRTEM and further confirms the incorporation of Cu into the Pt lattice and the formation of homogeneous alloys.

To examine the electrochemical activity and stability of the synthesized Pt_x_Cu (x = 1, 2, 3, 4) and pure Pt catalysts for IPAOR, cyclic voltammetry (CV) measurements were conducted over a potential window of 0–1.0 V vs. RHE for 100 cycles. The electrochemical surface areas (ECSAs) of the catalysts were calculated from CV curves in Figure S7, and all the subsequent current densities were normalized by their respective ECSAs. Figure 2d shows representative CV curves for PtCu in 1 M HClO_4_ with and without 1 M IPA, where a significant increase in oxidation current was observed in both the anodic and cathodic scans upon IPA addition. This indicates pronounced IPAOR activity compared to the acidic background. The IPAOR on PtCu exhibited an onset potential of approximately 0.30 V vs. RHE and achieved a peak current density of 10.1 mA/cm^2^_Pt_ at 0.8 V vs. RHE. Figure 2e summarizes the peak current densities of all samples after 100 cycles, showing that the Pt_x_Cu catalysts consistently outperformed pure Pt in activity. Stability was evaluated by monitoring the retention of peak current density over 100 cycles (Figure 2f and Figures S8–S12). Among the tested compositions, PtCu exhibited the best durability, maintaining over 80% of its initial activity throughout the test. Other catalysts including Pt_4_Cu, Pt_3_Cu, Pt_2_Cu, and pure Pt underwent a rapid initial activity drop to approximately 60–70% retention within the first five cycles, followed by stabilization. Tafel slope analysis (Figure S13) further supported the advantages of Cu alloying, with most alloyed catalysts exhibiting lower Tafel slopes than pure Pt, indicating faster reaction kinetics and enhanced electrocatalytic efficiency. The superior performance of PtCu over pure Pt can be attributed to alloying effects, where partial substitution of Pt with Cu not only reduces noble metal content but also enhances catalytic activity via geometric and electronic effects. These include lattice strain-induced rearrangement of Pt atoms and a downward shift in the Pt d-band center relative to the Fermi level, which optimizes the adsorption strength of reaction intermediates and promotes reaction kinetics [19].

**Figure 3:**
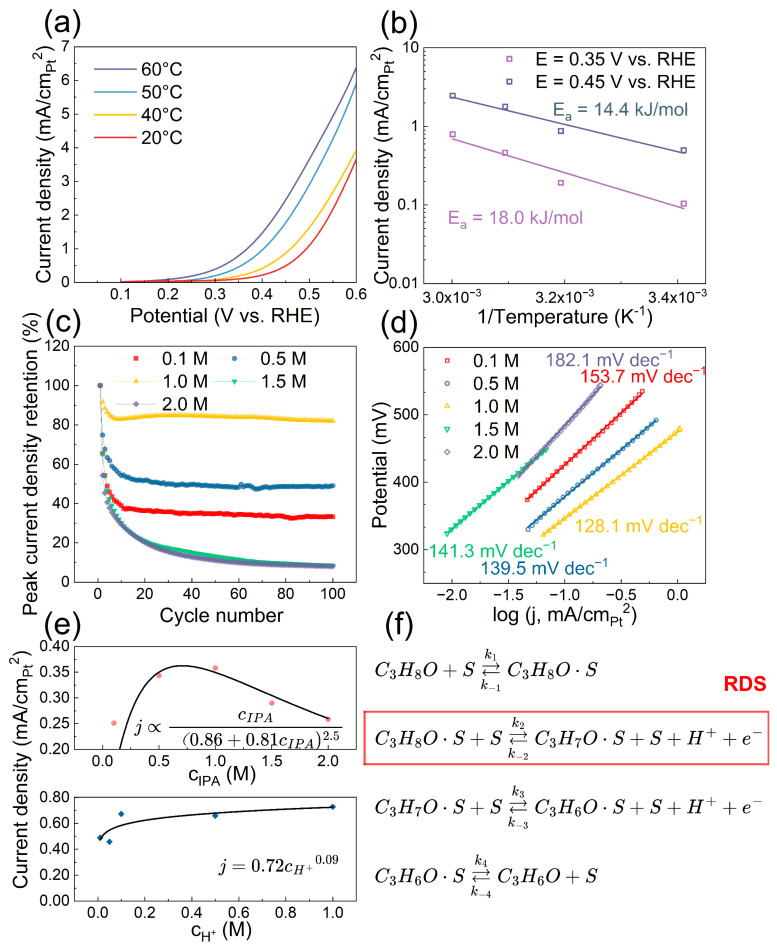
(**a**) LSV curves of PtCu in 1 M HClO_4_ + 1 M IPA from 0.1 to 0.6 V vs. RHE at 20, 40, 50 and 60 °C, respectively; (**b**) Arrhenius plots of PtCu-catalyzed IPAOR at 0.35 and 0.45 V vs. RHE; (**c**) Peak current density retentions of PtCu in 0.1, 0.5, 1.0, 1.5 and 2.0 M IPA, respectively; (**d**) Tafel plots of PtCu at 100th cycle in 0.1, 0.5, 1.0, 1.5 and 2.0 M IPA, respectively; (**e**) Current densities of PtCu at 0.4 V vs. RHE respect to various H^+^ and IPA concentrations, respectively; (**f**) Proposed reaction pathway of IPAOR under PtCu electrocatalysis (S: Pt active site on PtCu alloy).

Given the superior activity and stability of PtCu catalyst, it was selected for detailed kinetic studies of IPAOR under varying temperatures and concentrations. Temperature effects on IPAOR were examined using linear scan voltammetry (LSV) curves in 1 M HClO_4_ and 1 M IPA, measured from 0.1 to 0.6 V vs. RHE at 20, 40, 50, and 60 °C (Figure 3a). The current densities at 50 and 60 °C exhibited a significant increase compared to that of 20 °C, and the onset potential shifted negatively from 0.3 V vs. RHE to approximately 0.2 V vs. RHE when temperature rose to 60 °C. The apparent activation energy derived from the LSV curves was estimated to be 18.0 kJ/mol at 0.35 V vs. RHE and 14.4 kJ/mol at 0.45 V vs. RHE, confirming a low energy barrier for IPAOR on PtCu (Figure 3b).

The influence of IPA concentration (c_IPA_) on the catalytic performance of PtCu was systematically investigated. Figure 3c shows the peak current density retention over 100 cycles at various IPA concentrations in 1 M HClO_4_ (Figures S12, S14–S17). Catalyst stability was strongly dependent on c_IPA_, with the highest retention (82%) observed at 1 M IPA. As c_IPA_ increased from 0.1 M to 1 M, stability improved with retention rates of 33% at 0.1 M and 49% at 0.5 M. However, further increases in IPA concentration led to a marked decline in stability, with retention rates dropping to around 8% after 100 cycles for 1.5 and 2 M IPA. The Tafel slopes for 0.1, 0.5, 1, 1.5, and 2 M IPA are 153.7, 139.5, 128.1, 141.3, 182.1 mV/dec, respectively (Figure 3d). The lowest Tafel slope at 1 M IPA correlates with the highest observed stability, reinforcing its optimal concentration for performance in Figure 3c. This concentration-dependent behavior is attributed to changes in reaction kinetics and the rate-determining step (RDS), as discussed in the following mechanistic analysis.

To gain deeper mechanistic insight, we also explored the effect of H^+^ concentration (c_H_^+^) and proposed a potential mechanism to explain the observed concentration effects. Current densities for various c_H_^+^ and c_IPA_ at 0.4 V vs. RHE were extracted from the LSV curves (Figures S18 and S19), as this potential falls within the kinetics region according to the Tafel analysis. The plots of current densities versus c_H_^+^ and c_IPA_ at 0.4 V vs. RHE are depicted in Figure 3e. While c_IPA_ exhibited a volcano-shaped dependence, c_H_^+^ showed minimal influence on reaction rate, suggesting that proton concentration is not a major factor under these conditions. The current density-c_H_^+^ relationship was fitted to a power law model (Figure 3e), yielding a power value of 0.09, indicating no significant dependence on c_H_^+^. To rationalize this observation, a kinetic model was developed based on the proposed reaction pathway (Figure 3f), including four steps: (1) IPA adsorption, (2) first proton-coupled electron transfer (PCET), (3) second PCET, and (4) ACE desorption. The first PCET (step 2) was identified as the RDS, based on microkinetic modeling and theoretical rate law derivation (Equations (S1)–(S24)), which showed strong agreement with the experimentally observed rate behavior. Although previous literature has suggested that the ACE desorption (step 4) may be the RDS due to strong product adsorption and catalyst poisoning [12,16,20], our calculations demonstrate that step 2 as the RDS better aligns with our experimental trends, particularly the lack of c_H_^+^ dependence. The rate of IPAOR under this model can be expressed as:

(1) $$r=r_{2}=\frac{K_{1}k_{2}c_{IPA}}{{\left(1+K_{1}c_{IPA}\right)}^{2}}$$

where *K*_1_ is the equilibrium constant of step 1, and k_2_ is the forward rate constant of step 2. As shown in Figure 3e, fitting the current density versus *c_IPA_* to Equation (2) produced excellent agreement, further validating that the first proton transfer is the RDS. This mechanism also explains the system’s resistance to the poisoning effect.

**Figure 4:**
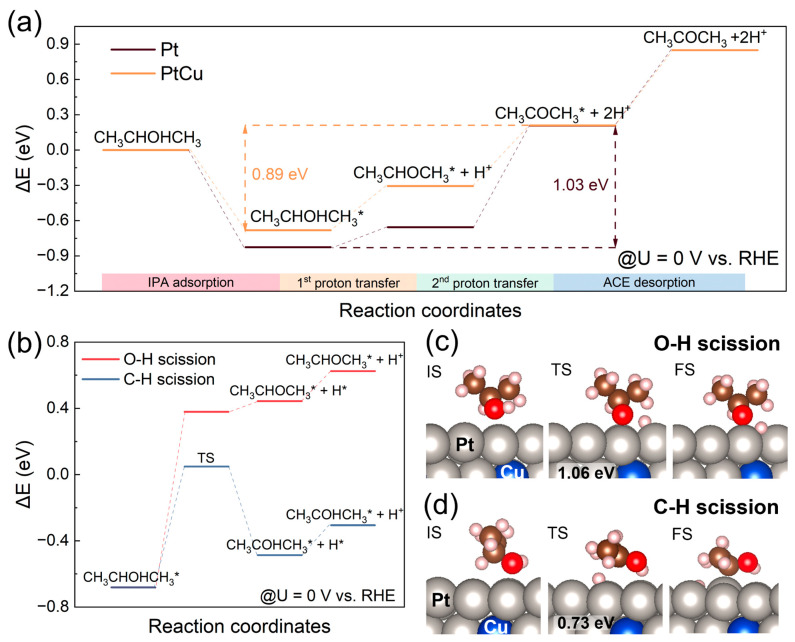
(**a**) Calculated free energy diagram for IPAOR on Pt and PtCu; (**b**) Calculated free energy diagram for two pathways of IPAOR on PtCu; (**c**) Adsorption configurations of initial state (IS), transition state (TS), and final state (FS) at PtCu surface for O-H scission pathway; (**d**) Adsorption configurations of IS, TS, and FS at PtCu surface for C-H scission pathway.

Based on experimental observations, two representative catalyst models of PtCu and Pt were constructed to investigate the intrinsic mechanism into IPAOR to ACE at U = 0 V vs. RHE using density functional theory (DFT) calculations. As illustrated in Figure 4a, the energy difference between adsorbed IPA and adsorbed ACE on the PtCu surface is 0.89 eV, which is lower than the corresponding energy difference on the Pt surface (1.03 eV). This reduced energy gap suggests a more thermodynamically favorable and balanced reaction pathway on PtCu, which may underline its superior catalytic performance relative to pure Pt. To gain mechanistic insight into IPAOR, we explored two possible reaction pathways on the PtCu surface and considered the transition state occurring before the first PCET step, which was identified as the RDS from kinetic studies. Two potential PCET pathways were proposed, distinguished by the initial bond scission step: the O-H scission pathway and the C-H scission pathway. In both pathways, IPA is first adsorbed onto the PtCu surface as CH_3_CHOHCH_3_*. This intermediate proceeds through a transition state (TS) to form CH_3_CHOCH_3_* or CH_3_COHCH_3_*, releasing an adsorbed H* that subsequently couples with an electron transfer and desorbs as H^+^. In the O-H scission pathway, CH_3_CHOHCH_3_* is converted to CH_3_CHOCH_3_*, but this pathway is energetically unfavorable, with a significant energy barrier of 1.06 eV and an unstable final state (CH_3_CHOCH_3_* + H*), rendering the pathway thermodynamically implausible (Figure 4b,c). In contrast, the C-H scission pathway is energetically favorable, featuring a lower energy barrier of 0.73 eV, leading to the formation of CH_3_COHCH_3_* as an intermediate. This species undergoes further oxidation to CH_3_COCH_3_*, which is finally desorbed as ACE (Figure 4b,d). These DFT results indicate that the C-H bond cleavage pathway is the preferred route for IPAOR on PtCu due to its lower energy barrier, offering a mechanistic basis for the enhanced activity observed experimentally. This theoretical finding corroborates experimental evidence and underscores the advantage of PtCu over pure Pt in suppressing reaction barriers and promoting efficient electro-oxidation.

**Figure 5:**
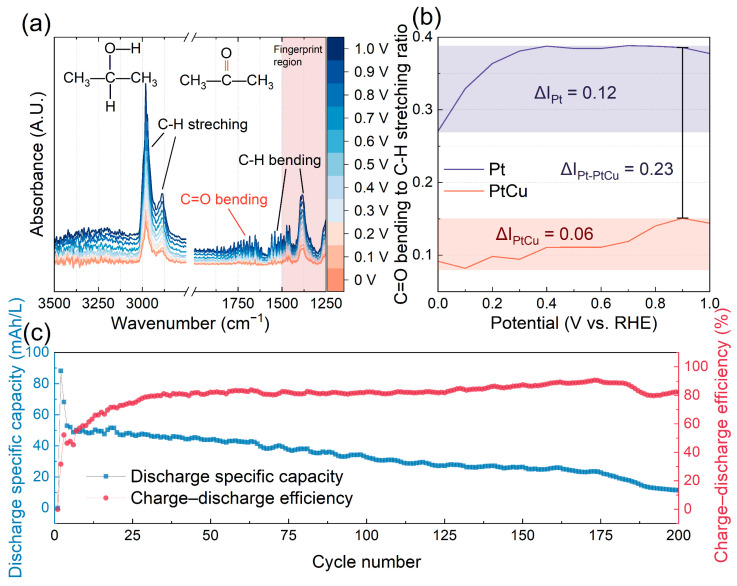
(**a**) In situ AIR-FTIR spectra of IPAOR on PtCu catalyst from 0 to 1.0 V vs. RHE; (**b**) The comparison of C=O bending to C-H stretching ratio profiles for PtCu and Pt; (**c**) Discharge specific capacity and charge–discharge efficiency of a demonstration of IPA redox chemistry in H-cell for 200 cycles.

In situ ATR-FTIR spectroscopy was employed to probe the surface species formed during IPAOR on PtCu. The spectra obtained from 0 to 1.0 V vs. RHE with potential and time control (Figure S20) are displayed in Figure 5a. The characteristic frequencies at 2980 and 2886 cm^−1^ for IPA and ACE are attributed to C-H stretching, while bands at approximately 1500 and 1387 cm^−1^ correspond to C-H bending [21,22]. As illustrated in Figure 5a, the intensities of both C-H stretching and bending increased with the applied potential, indicating an increase in surface coverage of IPA on the PtCu catalyst [23]. Additionally, the intensity of C=O feature bond at ~1682 cm^−1^ in ACE intensified gradually, confirming the progressive generation of oxidation products [21]. For comparison, ATR-FTIR spectra of pure Pt (Figure S21) show a significantly greater increase in the C=O signal with potential, suggesting higher ACE accumulation on Pt relative to PtCu. Quantitative analysis of the ratios of C=O bending to C-H stretching for PtCu and Pt at different potentials are presented in Figure 5b. PtCu demonstrated a significantly lower ACE coverage compared to Pt, with a difference (ΔI_Pt-PtCu_) of 0.23. Moreover, the potential-dependent increase in this ratio was smaller for PtCu (ΔI_PtCu_ = 0.06), indicating better resistance to poisoning. These spectroscopic findings reinforce the kinetic conclusion that ACE desorption is not the rate-determining step (RDS) on PtCu, and underscore the alloy’s improved anti-poisoning behavior relative to pure Pt.

An H-cell was assembled to evaluate the practical performance of IPA-based redox chemistry using PtCu catalyst. The anolyte composed of 50:50 volume ratio of IPA and ACE, while the catholyte was composed of a 50:50 mixture of V(V) and V(IV) species, separated by an anion exchange membrane (AEM) to facilitate anion transportation and maintain charge balance between the two compartments. The schematic of the H-cell and the corresponding electrode reactions are depicted in Figure 1b. Under PtCu catalysis, the H-cell was used to demonstrate the charge–discharge behavior of IPA within a voltage range of 0.2 V to 1.4 V. The specific capacity of this H-cell stabilized after the initial three cycles, reaching a value of 53 mAh/L and showing a charge–discharge efficiency of 80% (Figure 5c and Figure S22). Long-term stability testing showed that the PtCu-catalyzed IPA H-cell retained excellent cycling performance over 200 cycles, with only moderate capacity fading. In contrast, a previously reported IPA battery utilizing a pure Pt catalyst exhibited a significantly lower durability of only 50 cycles [13]. While this setup does not replicate the flow dynamics of a practical RFB, these results highlight the superior catalytic durability of PtCu and underscore its potential as a robust and cost-effective anode catalyst for IPA-based aqueous-organic redox chemistry for future aqueous-organic redox flow battery systems.

## 3. Materials and Methods

### 3.1. Materials and Chemicals

Carbon black (Vulcan XC 72R), carbon felt, carbon cloth (Panex-30-fabric-PW06) and anion exchange membrane (AEM, Fumasep-FAS-PET-130) were obtained from Fuel Cell Store (Bryan, TX, USA). Sodium borohydride (NaBH_4_, 98%), acetone (ACE, ACS grade), and vanadium(IV) sulfate oxide hydrate (VOSO_4_·xH_2_O, 99.9%, metals basis) were purchased from Thermo Fisher (Waltham, MA, USA). Copper(II) nitrate trihydrate (Cu(NO_3_)_2_·3H_2_O, 99%) and vanadium(V) oxide (V_2_O_5_, 98+%) were obtained from Acros (Morris Plains, NJ, USA). Potassium hexachloroplatinate(IV) (K_2_PtCl_6_, 98%), perchloric acid (HClO_4_, 70%), isopropanol (IPA, 99.5%), and sulfuric acid (H_2_SO_4_, 95.0–98.0%) were purchased from Sigma Aldrich (Saint Louis, MO, USA). Nafion dispersion (D521, 5%) was obtained from Ion Power (Tyrone, PA, USA). Argon gas (Ar, 99.999%) was supplied by Linde (Danbury, CT, USA).

### 3.2. Synthesis and Characterization of Pt_x_Cu Alloy Catalysts

Pt_x_Cu (x = 1, 2, 3, 4) and Pt nanoparticle catalysts were synthesized by controlling the molar ratio of K_2_PtCl_6_ to Cu(NO_3_)_2_·3H_2_O precursors. Specifically, 0.1 mmol K_2_PtCl_6_ and the corresponding 0.1/x mmol Cu(NO_3_)_2_·3H_2_O were dissolved in 19 mL of DI water. Subsequently, 78 mg carbon black was added to the solution, and the mixture was stirred at 500 rpm for 30 min under argon gas flow. A freshly prepared solution of 150 mg NaBH_4_ in 1 mL DI water was then introduced to initiate reduction, and the reaction was maintained under argon protection for 1 h. The resulting product was collected by centrifugation, washed three times with DI water, and vacuum-dried at 70 °C overnight.

The synthesized catalysts were characterized using multiple techniques. X-ray diffraction (XRD) was performed on a Rigaku MiniFlex II (Rigaku, Tokyo, Japan) (10°–90° scan range) for phase information. Transmission electron microscopy (TEM) images were acquired using a Hitachi HT7800 (Hitachi, Marunouchi, Japan), and high-resolution TEM (HRTEM) was conducted with a Hitachi H9500 (Hitachi, Marunouchi, Japan) to characterize lattice structures. Elemental compositions were determined by energy-dispersive X-ray spectroscopy (EDS) using a Zeiss Gemini500 (Zeiss, Oberkochen, Germany) field emission scanning electron microscope (SEM). Thermogravimetric analysis (TGA) was carried out on a Shimadzu TGA-50H (Shimadzu, Kyoto, Japan), where samples were heated from room temperature to 110 °C, held at the temperature for 30 min for moisture removal, then ramped to 800 °C at 10 °C/min with a 10 min hold for metal content analysis.

### 3.3. Electrochemical Testing of IPAOR on Pt_x_Cu Catalysts

Electrodes for half-cell evaluation of IPAOR on Pt_x_Cu catalysts were prepared by dispersing 3 mg catalyst in a 3 mL solution composed of IPA, DI water, and Nafion dispersion in a volumetric ratio of 250:250:1. The resulting ink was sonicated for 30 min to ensure uniform dispersion. A 10 μL aliquot of the ink was drop-cast onto a glassy carbon rotating disk electrode (RDE) with a diameter of 5 mm and dried using a hot air stream while rotating at 250 rpm to ensure uniform film formation.

The prepared electrode was used as the working electrode (WE), with a platinum coil serving as the counter electrode (CE) and a reversible hydrogen electrode (RHE, HydroFlex) as the reference electrode (RE). Electrochemical measurements were conducted in a three-electrode configuration using a CHI 760d electrochemical workstation (CH Instrument, Austin, TX, USA) under an argon atmosphere. Cyclic voltammetry (CV) and linear scan voltammetry (LSV) were performed at a scan rate of 50 mV/s in electrolytes containing specified concentrations of HClO_4_ and IPA.

In situ attenuated total reflectance-Fourier transform infrared spectroscopy (ATR-FTIR) was conducted using a Nicolet 6700 FTIR spectrometer (Thermo Electron Corporation, Madison, WI, USA) equipped with a VeeMAX III ATR accessory (PIKE Technologies, Madison, WI, USA). Catalyst ink was drop-cast onto carbon cloth, which served as the WE in the ATR cell. The ATR crystal functioned as the current collector, allowing infrared transparency during measurements. A multipotential step method was employed, applying potentials from 0 to 1.0 V vs. RHE in 0.1 V increments with a dwell time of 60 s at each step.

### 3.4. Assembly and Electrochemical Testing of IPA/ACE-Based H-Cell

To validate the feasibility of the IPA/ACE redox couple, a two-electrode H-cell configuration was assembled and tested. The PtCu catalyst ink containing 2 mg catalyst was drop-cast onto a 1 cm^2^ carbon felt, which served as the negative electrode. A blank carbon felt was used as the positive electrode. The anolyte consisted of 15 mL of aqueous solution containing 0.5 M H_2_SO_4_, 0.5 M IPA, and 0.5 M ACE. The catholyte comprised 15 mL of 0.5 M H_2_SO_4_, 0.02 M VOSO_4_, and 0.01 M V_2_O_5_. An AEM was placed between the two compartments to ensure ionic conductivity and charge balance.

Galvanostatic charge–discharge (GCD) tests were performed using a Neware battery testing system to assess the electrochemical reversibility and cycling stability of the IPA/ACE system. The cell was operated at a constant current density of 0.3 mA/cm^2^ within a voltage window of 0.2 to 1.4 V. Long-term performance was evaluated over 200 cycles to examine the practical viability of IPA/ACE chemistry.

### 3.5. Density Functional Theory (DFT) Calculations

DFT calculations were performed using the Vienna ab initio Simulation Package (VASP, Version 5.4.3) [24,25]. The core electrons were approximated using the projector augmented wave (PAW) method [26], and the generalized gradient approximation Perdew–Burke–Ernzerhof (GGA-PBE) functional was used to account for the electron exchange–correlation effects [27]. Furthermore, Grimme’s DFT-D3 correction term was used for the van der Waals dispersion [28]. All DFT calculations employ the Methfessel and Paxton method [29] to describe partial orbital occupancies near the Fermi level, with the sigma value of 0.2 eV.

The bulk structures of face-centered cubic Pt and Pt_3_Cu were relaxed using a 16 × 16 × 16 *k*-point mesh based on the Monkhorst–Pack scheme [30], with a plane-wave cutoff energy of 400 eV, resulting in lattice parameters of 3.94 Å for Pt, and 3.88 Å and 3.56 Å for Pt_3_Cu, respectively. The close-packed (111) surfaces cleaved from Pt and Pt_3_Cu bulk were used to model the IPAOR. The *p*(3 × 3) Pt and Pt_3_Cu slab cells consist of four layers with the bottom two layers fixed using the lattice parameters of respective Pt and Pt_3_Cu bulks. A Pt monolayer overlayer was placed on the Pt_3_Cu slab to represent a surface-enriched Pt configuration due to leaching out of Cu on the surface in acidic condition, and a 15 Å vacuum layer was included normal to the slab to avoid spurious interactions between periodic images. A 4 × 4 × 1 *k*-point mesh was used for surface calculations, and dipole corrections were applied when adsorbates were present.

Reaction pathways for the first C-H and O-H bond scission steps in IPAOR were investigated using the climbing image nudged elastic band (CI-NEB) method [31] in combination with the dimer method [32]. Transition states were verified by vibrational frequency analysis, ensuring the presence of a single imaginary mode.

Free energy changes for the electro-oxidation steps were estimated using the computational hydrogen electrode (CHE) model developed by Nørskov and coworkers [33], which equate the chemical potential of a proton–electron pair ($\mu \left(H^{+}+e^{-}\right)$) to that of ½ H_2_ in the gas phase:

(2) $$\mu \left(H^{+}+e^{-}\right)=\;\frac{1}{2}\mu \left(H_{2\left(g\right)}\right)-eU$$

## 4. Conclusions

In summary, we successfully synthesized and characterized a series of Pt_x_Cu alloy catalysts with varying Pt:Cu ratios and evaluated their performance for IPAOR. Among the compositions, PtCu exhibited the highest stability and the lowest Tafel slope, identifying it as the optimal catalyst. Electrochemical studies under varying temperatures and concentrations revealed a low apparent activation energy of 14.4 kJ/mol at 0.45 V vs. RHE, indicating favorable IPAOR kinetics. Stability testing as a function of IPA concentration showed that 1 M IPA provides the best performance, while higher concentrations adversely affected catalyst stability. Kinetic analysis and mechanistic studies across various IPA and H^+^ concentrations suggested that the first PCET step is the RDS, rather than ACE desorption, for IPAOR on PtCu. This mechanistic behavior likely contributes to the enhanced stability of PtCu. DFT calculations further supported this conclusion, revealing that the IPAOR pathways are more balanced on PtCu than on Pt and suggesting an improvement in the reaction kinetics. The first PCET during IPAOR on the PtCu surface preferentially follows the C-H scission pathway due to its lower activation energy (0.73 eV) compared to the O-H scission pathway (1.01 eV). In situ ATR-FTIR spectroscopy confirmed lower surface accumulation of ACE species on PtCu relative to pure Pt, reinforcing its improved resistance to catalyst poisoning. Finally, an H-cell RFB employing PtCu as the anode catalyst and IPA/ACE as the redox couple demonstrated excellent cycling stability over 200 cycles. Although the H-cell does not mimic full redox flow battery conditions, these results highlight the feasibility of IPA/PtCu chemistry and its potential as a cost-effective, durable platform for future aqueous-organic redox flow energy storage systems. Further optimization and scale-up efforts will be critical to enhancing specific capacity and efficiency, enabling practical deployment of IPA-based energy storage technologies.

## Data Availability

The original contributions presented in this study are included in the article/Supplementary Material. Further inquiries can be directed to the corresponding author.

## Supplementary Materials

The following supporting information can be downloaded at: https://www.mdpi.com/article/10.3390/molecules30194027/s1, HRTEM images, CV curves of Pt_x_Cu samples, Calculation of reaction kinetics.

## References

[1] Arutyunov V.S., Lisichkin G.V. (2017). Energy resources of the 21st century: Problems and forecasts. Can renewable energy sources replace fossil fuels. Russ. Chem. Rev..

[2] Chu S., Majumdar A. (2012). Opportunities and challenges for a sustainable energy future. Nature.

[3] Pahlevaninezhad M., Leung P., Velasco P.Q., Pahlevani M., Walsh F.C., Roberts E.P., de León C.P. (2021). A nonaqueous organic redox flow battery using multi-electron quinone molecules. J. Power Sources.

[4] Jin S., Jing Y., Kwabi D.G., Ji Y., Tong L., De Porcellinis D., Goulet M.-A., Pollack D.A., Gordon R.G., Aziz M.J. (2019). A water-miscible quinone flow battery with high volumetric capacity and energy density. ACS Energy Lett..

[5] Cao J., Tian J., Xu J., Wang Y. (2020). Organic flow batteries: Recent progress and perspectives. Energy Fuels.

[6] Nutting J.E., Rafiee M., Stahl S.S. (2018). Tetramethylpiperidine N-oxyl (TEMPO), phthalimide N-oxyl (PINO), and related N-oxyl species: Electrochemical properties and their use in electrocatalytic reactions. Chem. Rev..

[7] Yang Z., Tong L., Tabor D.P., Beh E.S., Goulet M.A., De Porcellinis D., Aspuru-Guzik A., Gordon R.G., Aziz M.J. (2018). Alkaline benzoquinone aqueous flow battery for large-scale storage of electrical energy. Adv. Energy Mater..

[8] Hoober-Burkhardt L., Krishnamoorthy S., Yang B., Murali A., Nirmalchandar A., Prakash G.S., Narayanan S. (2017). A new Michael-reaction-resistant benzoquinone for aqueous organic redox flow batteries. J. Electrochem. Soc..

[9] Huskinson B., Marshak M.P., Suh C., Er S., Gerhardt M.R., Galvin C.J., Chen X., Aspuru-Guzik A., Gordon R.G., Aziz M.J. (2014). A metal-free organic–inorganic aqueous flow battery. Nature.

[10] Yang G., Zhu Y., Hao Z., Zhang Q., Lu Y., Yan Z., Chen J. (2024). An Aqueous All-Quinone-Based Redox Flow Battery Employing Neutral Electrolyte. Adv. Energy Mater..

[11] Quinn A.H., Bu L.P., Brushett F.R. (2022). Investigation of the Isopropanol-Acetone Redox Couple for Rechargeable Liquid Organic Fuel Cells. Electrochemical Society Meeting Abstracts 242.

[12] Hauenstein P., Seeberger D., Wasserscheid P., Thiele S. (2020). High performance direct organic fuel cell using the acetone/isopropanol liquid organic hydrogen carrier system. Electrochem. Commun..

[13] Tang J., Li J., Pishva P., Xie R., Peng Z. (2023). Aqueous, Rechargeable Liquid Organic Hydrogen Carrier Battery for High-Capacity, Safe Energy Storage. ACS Energy Lett..

[14] Sievi G., Geburtig D., Skeledzic T., Bösmann A., Preuster P., Brummel O., Waidhas F., Montero M.A., Khanipour P., Katsounaros I. (2019). Towards an efficient liquid organic hydrogen carrier fuel cell concept. Energy Environ. Sci..

[15] Tang J., Xie R., Pishva P., Shen X., Zhu Y., Peng Z. (2024). Recent progress and perspectives of liquid organic hydrogen carrier electrochemistry for energy applications. J. Mater. Chem. A.

[16] Qi Z., Kaufman A. (2002). Performance of 2-propanol in direct-oxidation fuel cells. J. Power Sources.

[17] Kim H., Kim D., Son J.-I., Jang S., Chung D.Y. (2024). Mixed Potential Driven Self-Cleaning Strategy in Direct Isopropanol Fuel Cells. ACS Catal..

[18] Hu B., Yuan J., Zhang J., Shu Q., Guan D., Yang G., Zhou W., Shao Z. (2021). High activity and durability of a Pt–Cu–Co ternary alloy electrocatalyst and its large-scale preparation for practical proton exchange membrane fuel cells. Compos. Part B Eng..

[19] Yao P., Cao J., Ruan M., Song P., Gong X., Han C., Xu W. (2022). Engineering PtCu nanoparticles for a highly efficient methanol electro-oxidation reaction. Faraday Discuss..

[20] Cheng Y., Liu Y., Cao D., Wang G., Gao Y. (2011). Effects of acetone on electrooxidation of 2-propanol in alkaline medium on the Pd/Ni-foam electrode. J. Power Sources.

[21] Mekazni D.S., Arán-Ais R.M., Herrero E., Feliu J.M. (2023). On the oxidation of isopropanol on platinum single crystal electrodes. A detailed voltammetric and FTIR study. J. Power Sources.

[22] Waidhas F., Haschke S., Khanipour P., Fromm L., Go A., Bachmann J., Katsounaros I., Mayrhofer K.J., Brummel O., Libuda J.R. (2020). Secondary alcohols as rechargeable electrofuels: Electrooxidation of isopropyl alcohol at Pt electrodes. ACS Catal..

[23] Tang C., Wei C., Fang Y., Liu B., Song X., Bian Z., Yin X., Wang H., Liu Z., Wang G. (2024). Electrocatalytic hydrogenation of acetonitrile to ethylamine in acid. Nat. Commun..

[24] Kresse G., Hafner J. (1993). Ab initio Molecular Dynamics for Open-Shell Transition Metals. Phys. Rev. B.

[25] Kresse G., Furthmuller J. (1996). Efficiency of Ab-initio Total Energy Calculations for Metals and Semiconductors Using a Plane-Wave Basis Set. Comput. Mater. Sci..

[26] Blöchl P.E. (1994). Projector Augmented-Wave Method. Phys. Rev. B.

[27] Perdew J.P., Burke K., Ernzerhof M. (1996). Generalized Gradient Approximation Made Simple. Phys. Rev. Lett..

[28] Grimme S., Antony J., Ehrlich S., Krieg H. (2010). A consistent and accurate ab initio parametrization of density functional dispersion correction (DFT-D) for the 94 elements H-Pu. J. Chem. Phys..

[29] Methfessel M., Paxton A.T. (1989). High-precision sampling for Brillouin-zone integration in metals. Phys. Rev. B.

[30] Monkhorst H.J., Pack J.D. (1976). Special points for Brillouin-zone integrations. Phys. Rev. B.

[31] Henkelman G., Uberuaga B.P., Jónsson H. (2000). A climbing image nudged elastic band method for finding saddle points and minimum energy paths. J. Chem. Phys..

[32] Henkelman G., Jónsson H. (1999). A dimer method for finding saddle points on high dimensional potential surfaces using only first derivatives. J. Chem. Phys..

[33] Kitchin J.R., Nørskov J.K., Barteau M.A., Chen J.G. (2004). Role of Strain and Ligand Effects in the Modification of the Electronic and Chemical Properties of Bimetallic Surfaces. Phys. Rev. Lett..

