# Supplementary material for: Isopropanol Electro-Oxidation on PtCu Alloys for Aqueous Organic Redox Chemistry Toward Energy Storage
Source: Molecules. 2025 Oct 9;30(19):4027. doi: 10.3390/molecules30194027 (PMC12525896; doi:10.3390/molecules30194027)
Supplement: Supplementary file 1 [file molecules-30-04027-s001.zip › molecules-3861812-supplementary.pdf]

Calculation of the theoretical capacity of IPA

For a certain electrode material, the theoretical capacity is:

$$C_0 = \frac{nF}{3.6M}$$

Where M is the molar mass of the active material, n is the number of electrons gained and lost in the reaction and F is the Faraday constant.

$$C_{0-IPA} = \frac{2 \times 96485}{3.6 \times 60} = 893\ mAh/g$$

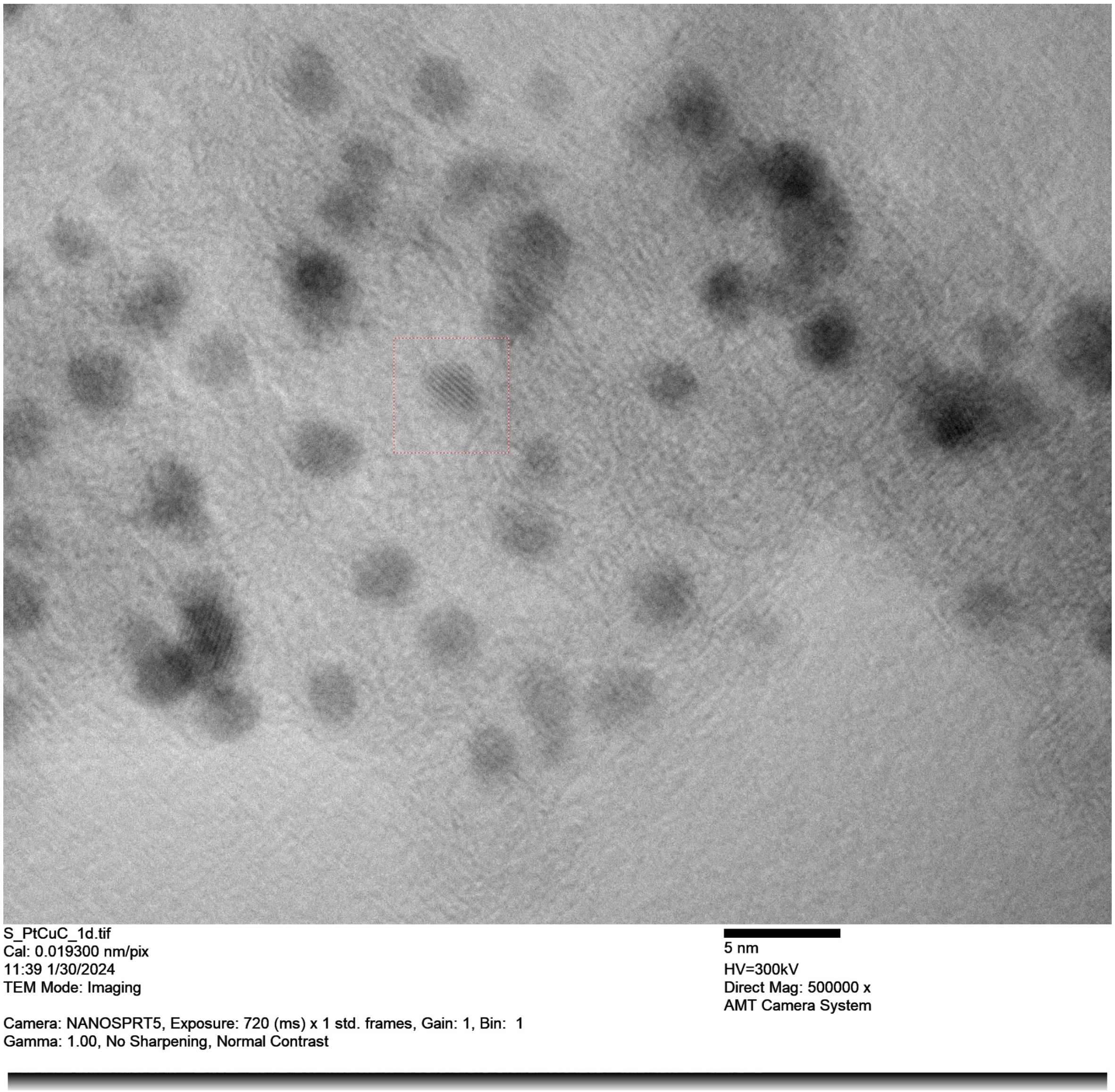

Figure S1. HRTEM image of PtCu sample.

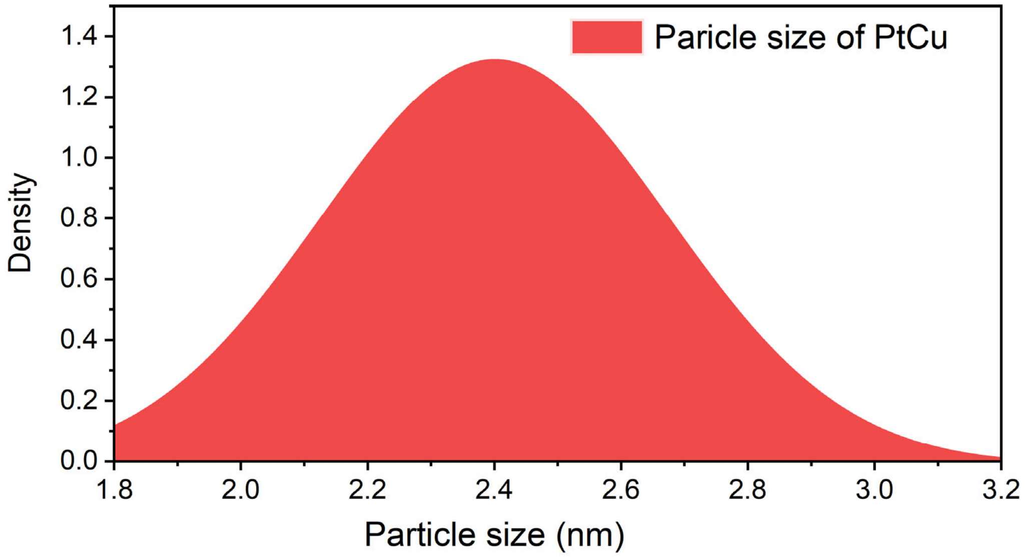

Figure S2. Particle size distribution of PtCu sample.

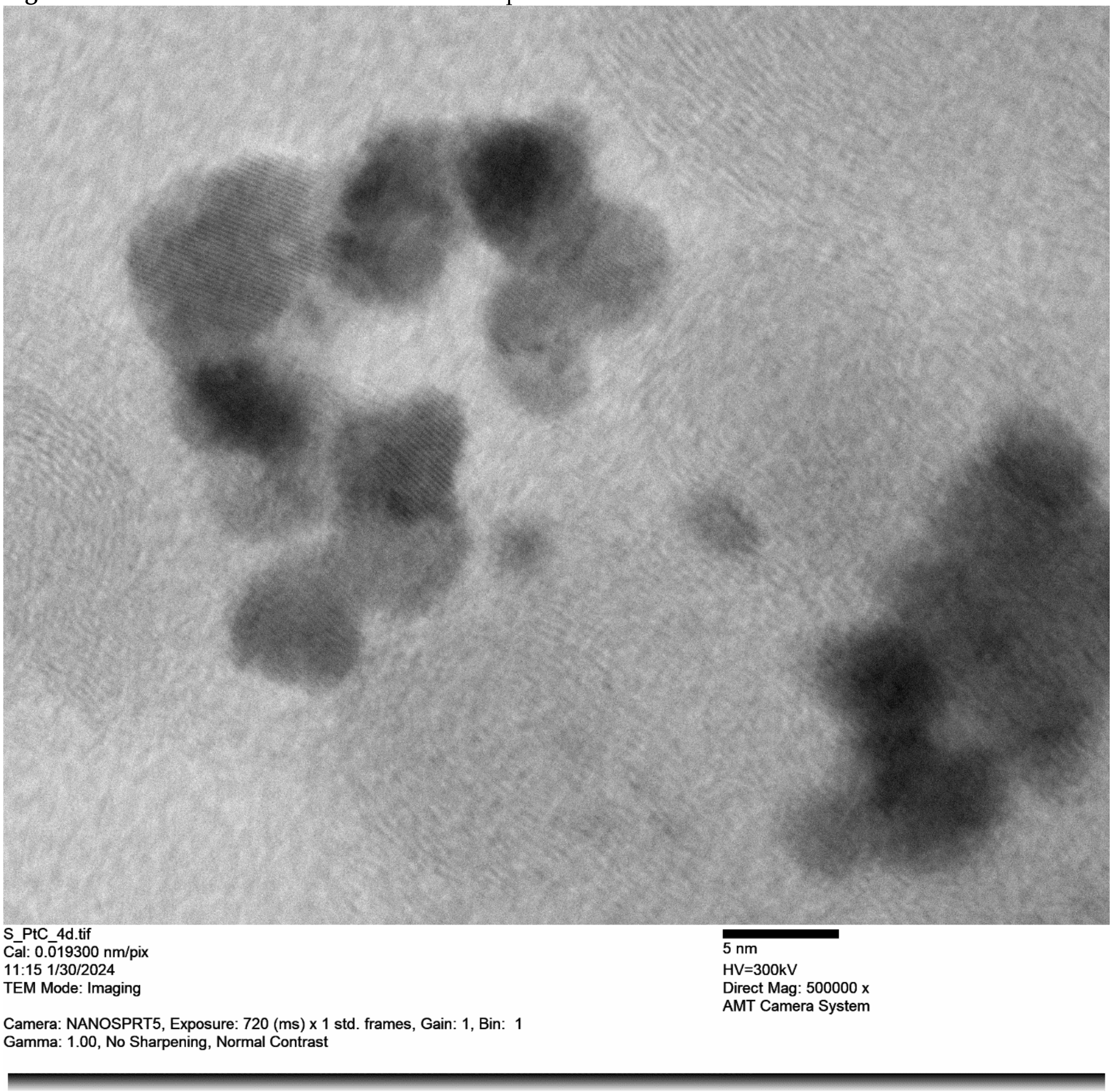

Figure S3. HRTEM image of Pt sample.

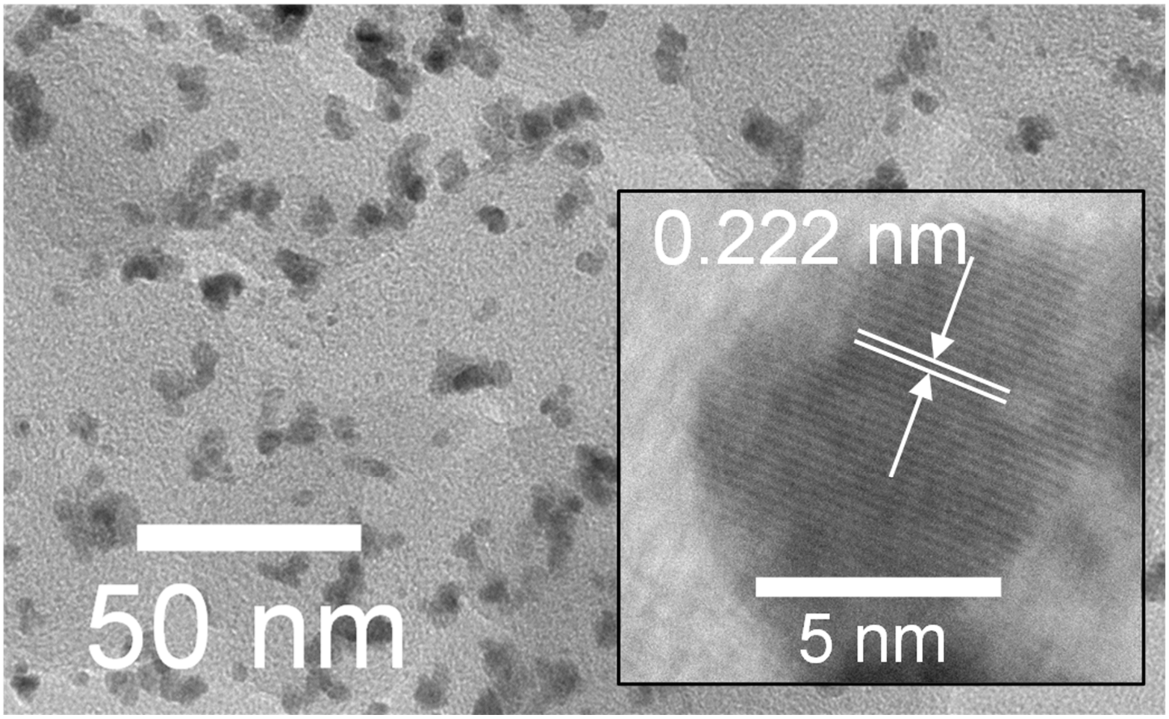

Figure S4. TEM and HRTEM images of Pt sample.

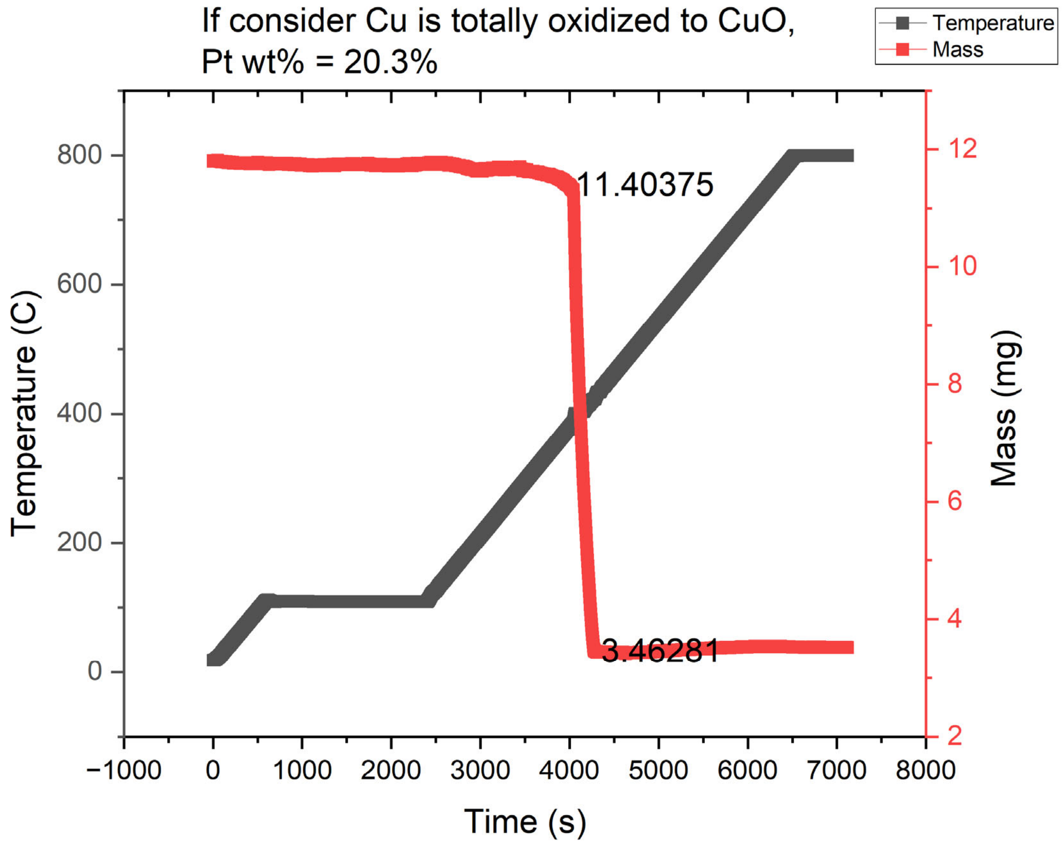

Figure S5. TGA results of PtCu sample.

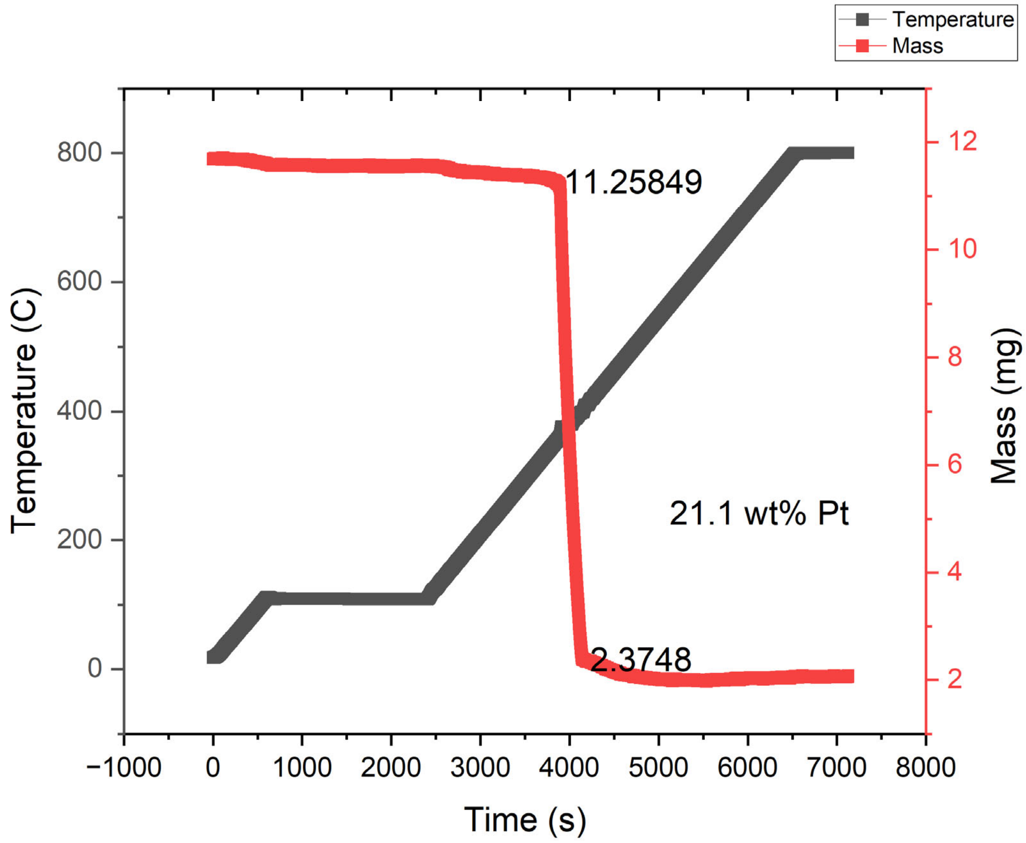

Figure S6. TGA results of Pt sample.

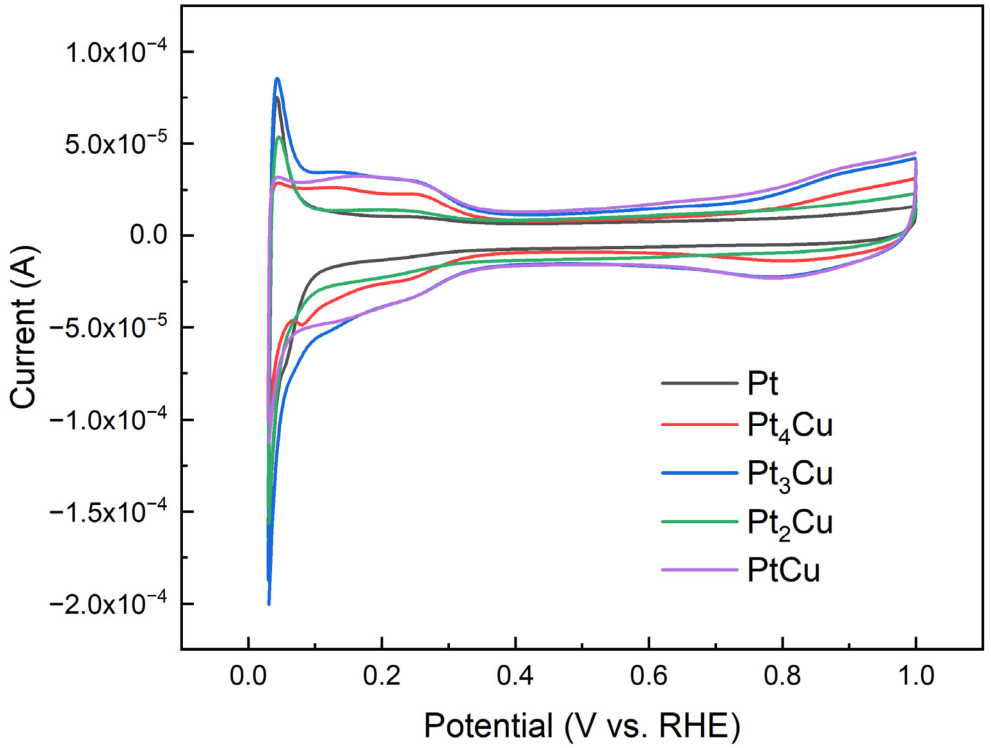

Figure S7. CV curves of Pt_xCu (x = 1, 2, 3, 4) and pure Pt samples in 1 M $HClO_4$. ECSA: Pt: 0.302 cm^2; Pt_4Cu: 0.533 cm^2; Pt_3Cu: 0.733 cm^2; Pt_2Cu: 0.325 cm^2; PtCu: 0.621 cm^2.

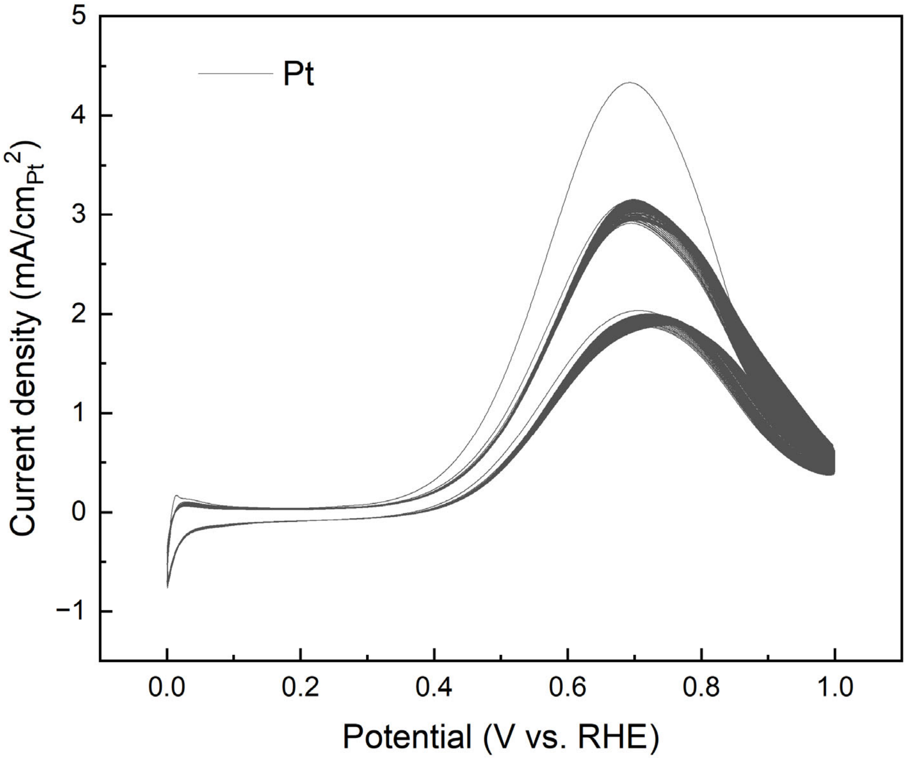

Figure S8. CV curves of Pt in 1 M $HClO_4$ + 1 M IPA for 100 cycles.

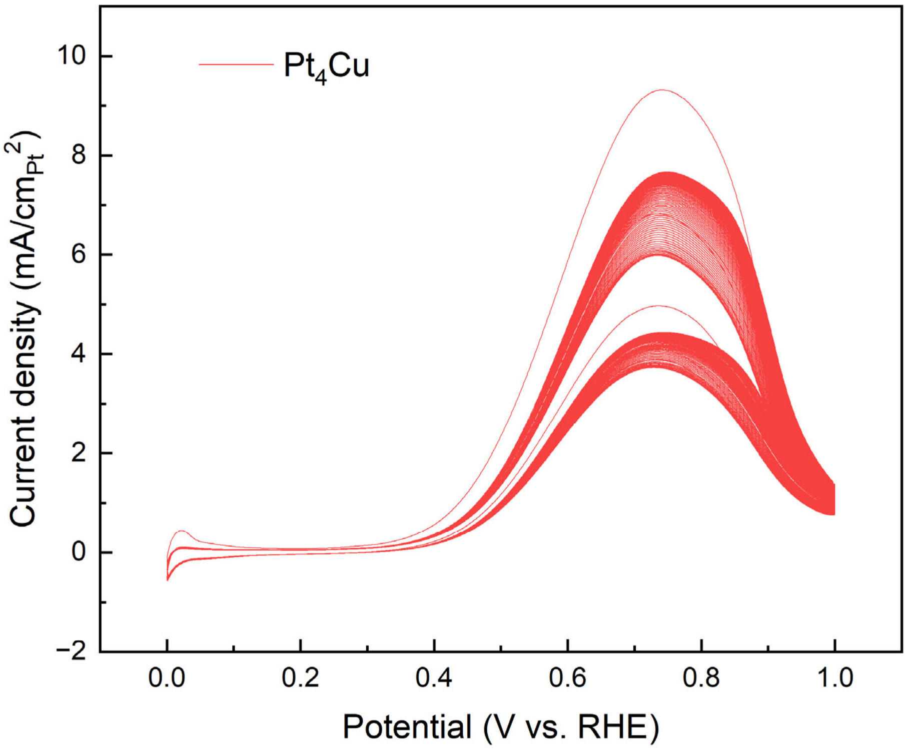

Figure S9. CV curves of Pt_4Cu in 1 M $HClO_4$ + 1 M IPA for 100 cycles.

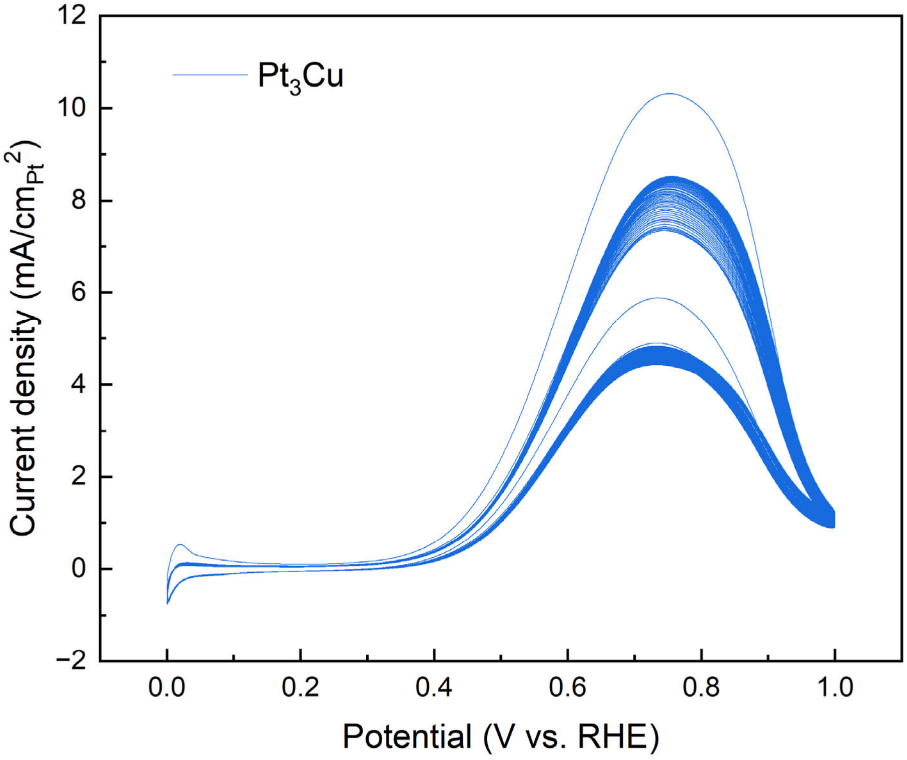

Figure S10. CV curves of Pt_3Cu in 1 M $HClO_4$ + 1 M IPA for 100 cycles.

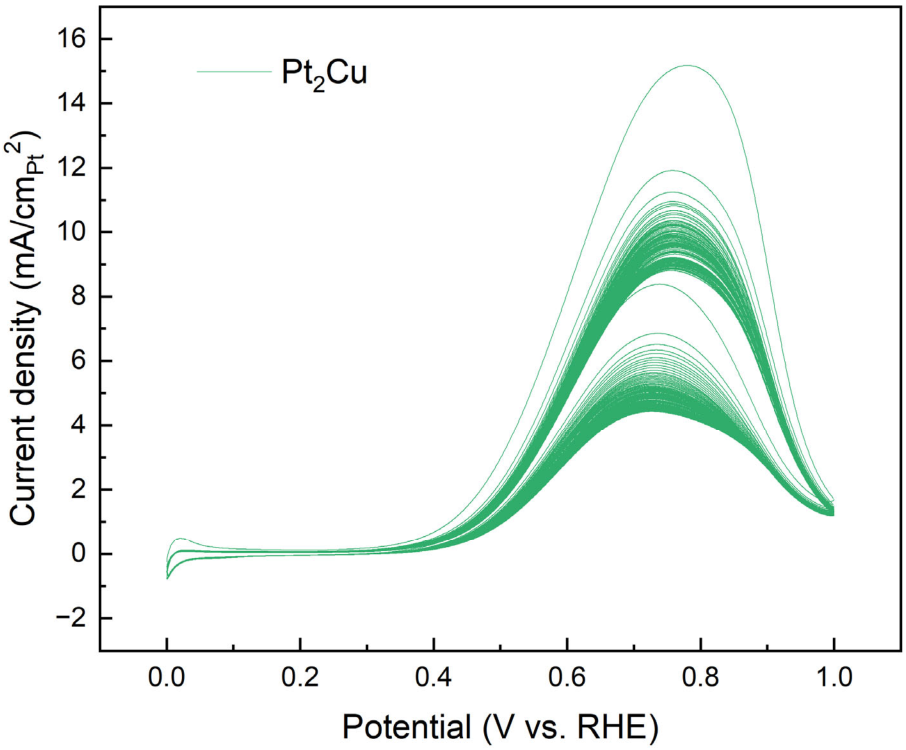

Figure S11. CV curves of Pt_2Cu in 1 M $HClO_4$ + 1 M IPA for 100 cycles.

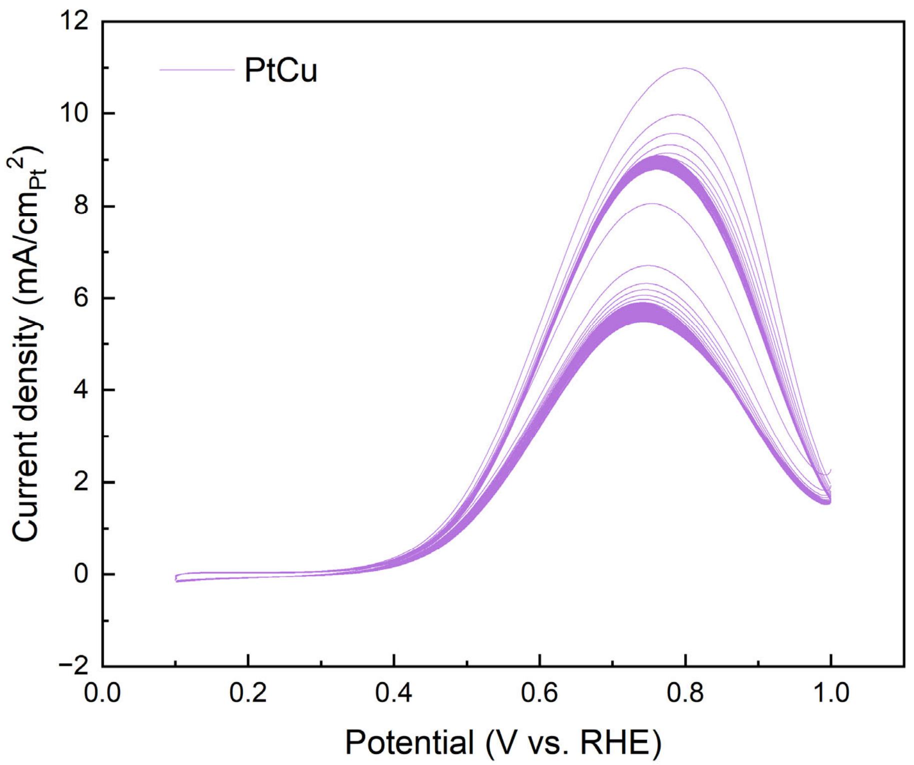

Figure S12. CV curves of PtCu in 1 M $HClO_4$ + 1 M IPA for 100 cycles.

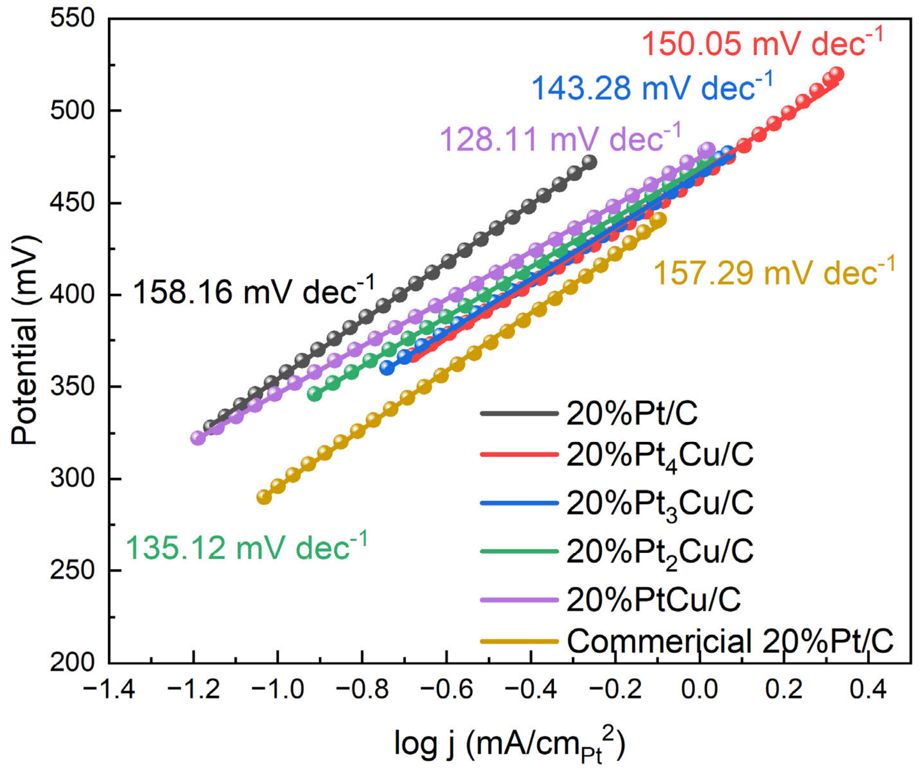

Figure S13. Tafel plots of Pt_xCu samples (x = 1, 2, 3, 4) and Pt in 1 M $HClO_4$ + 1 M IPA at 100th cycle.

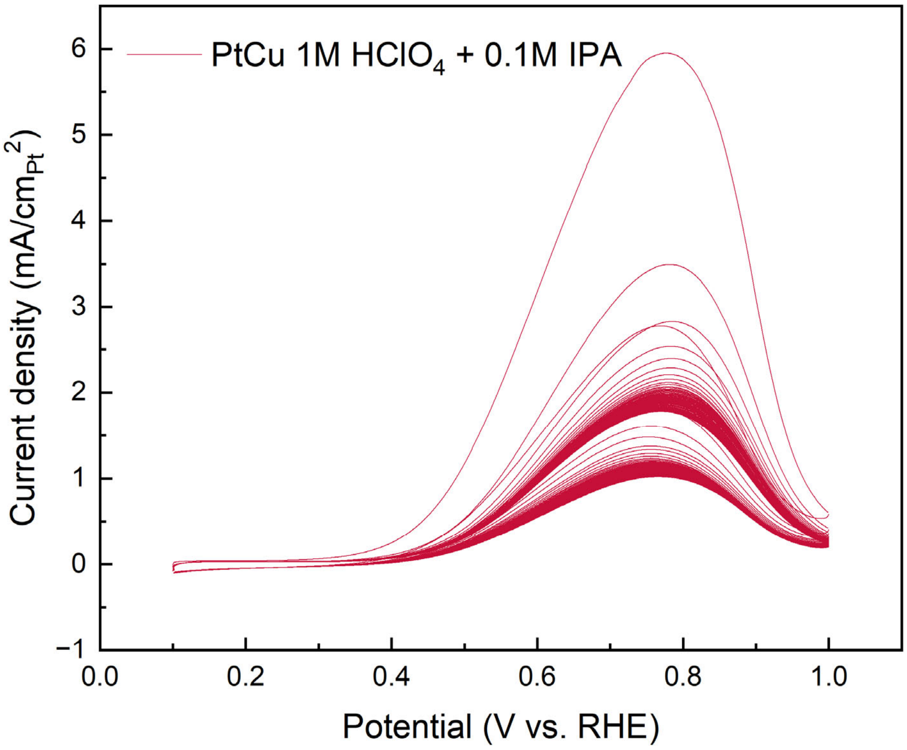

Figure S14. CV curves of PtCu in 1 M $HClO_4$ + 0.1 M IPA for 100 cycles.

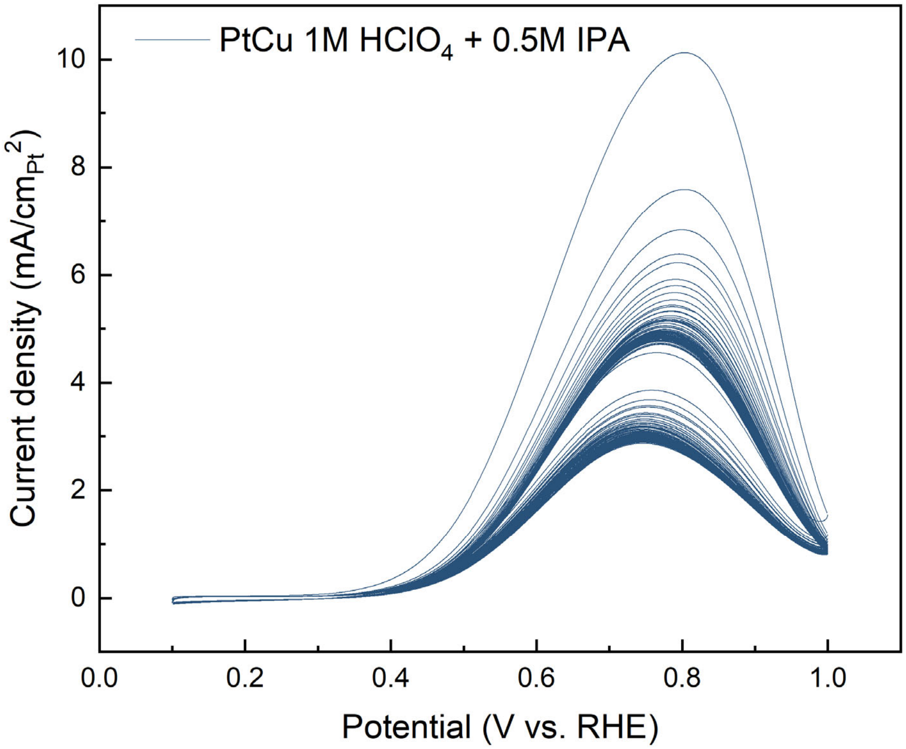

Figure S15. CV curves of PtCu in 1 M $HClO_4$ + 0.5 M IPA for 100 cycles.

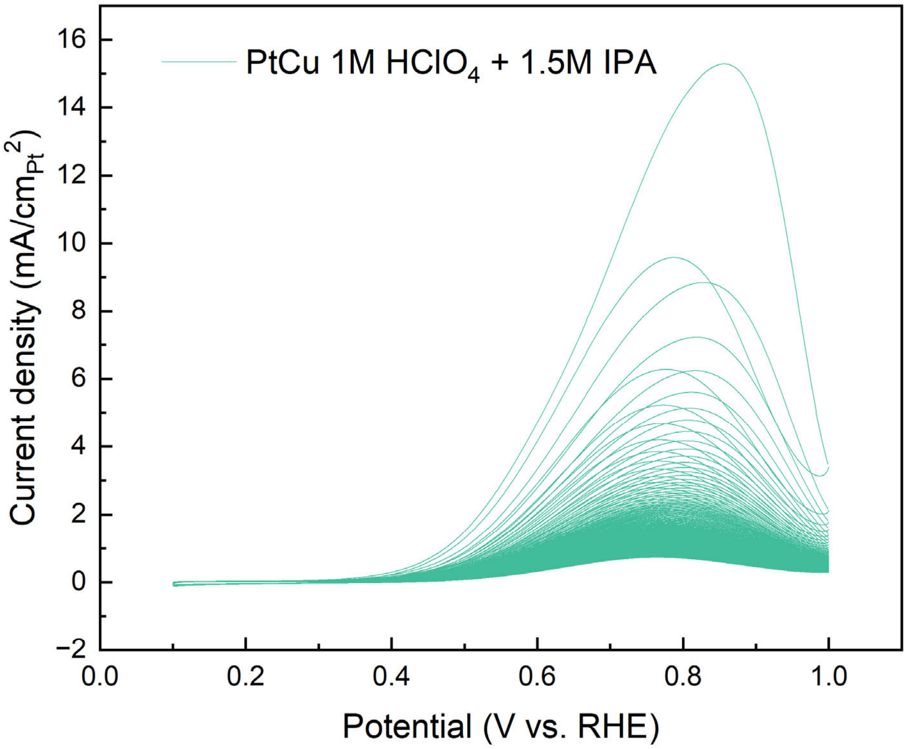

Figure S16. CV curves of PtCu in 1 M $HClO_4$ + 1.5 M IPA for 100 cycles.

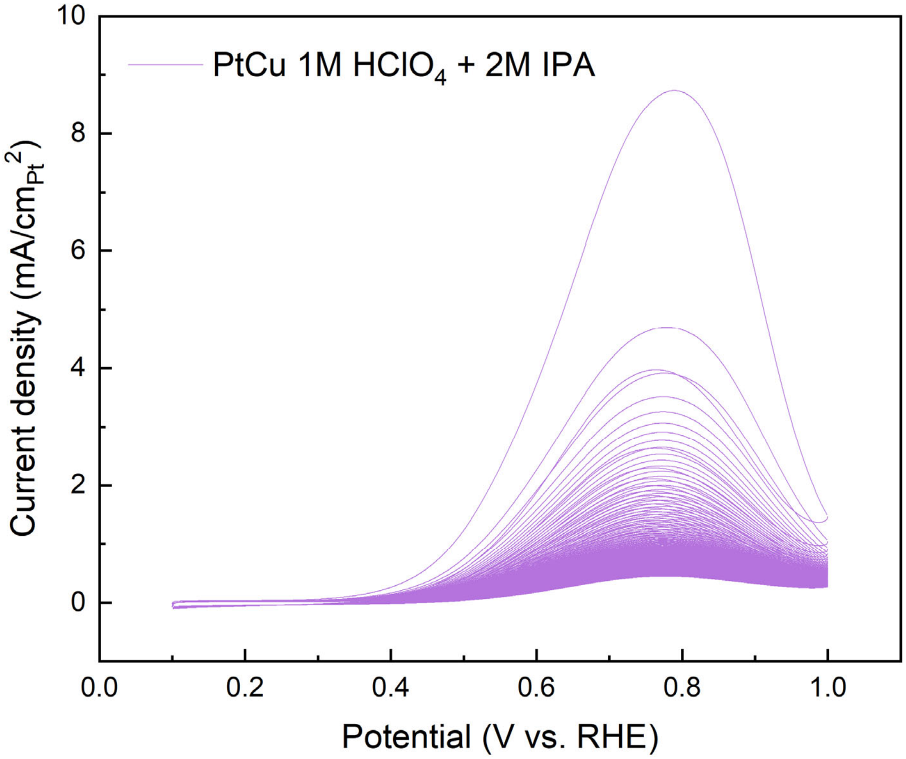

Figure S17. CV curves of PtCu in 1 M $HClO_4$ + 2 M IPA for 100 cycles.

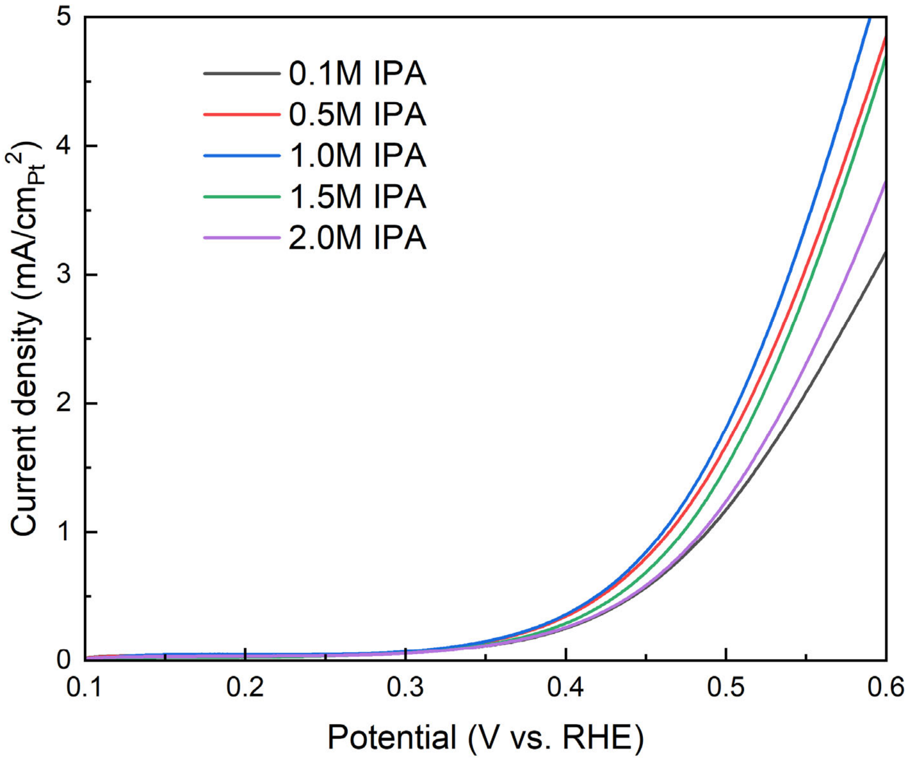

Figure S18. LSV curves of PtCu in 1 M $HClO_4$ + 0.1, 0.5, 1.0, 1.5, and 2.0 M IPA.

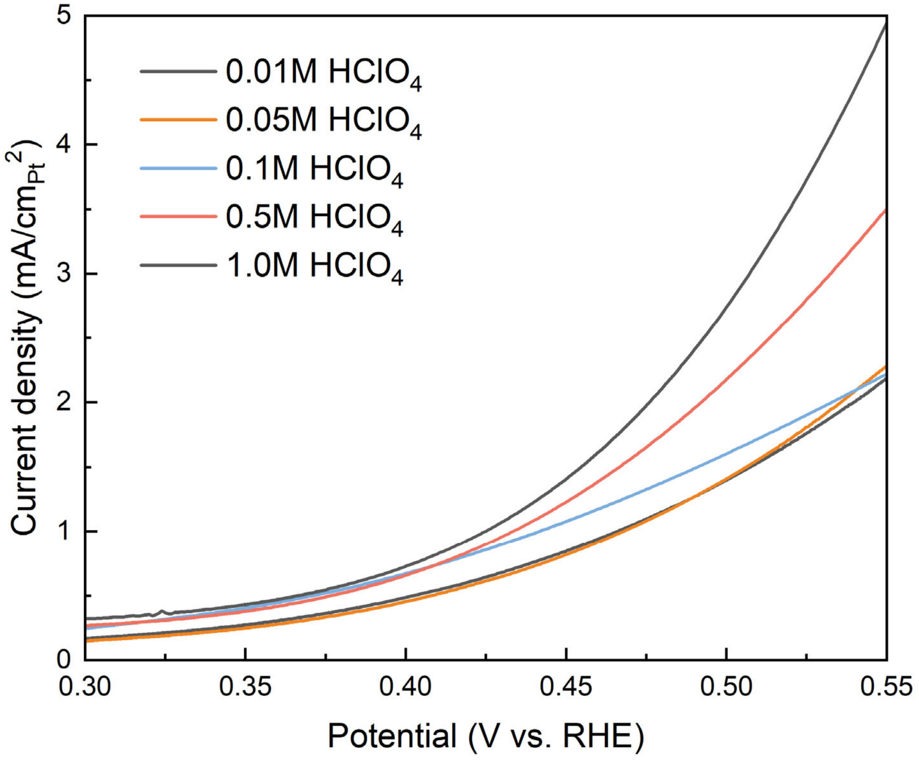

Figure S19. LSV curves of PtCu in 0.01, 0.05, 0.1, 0.5, and 1 M $HClO_4$ + 1 M IPA.

Reaction rate calculations

$$C_3H_8O + S \underset{k_{-1}}{\overset{k_1}{\rightleftarrows}} C_3H_8O \cdot S \quad (S1)$$

$$C_3H_8O \cdot S + S \underset{k_{-2}}{\overset{k_2}{\rightleftarrows}} C_3H_7O \cdot S + S + H^+ + e^- \quad (S2)$$

$$C_3H_7O \cdot S + S \underset{k_{-3}}{\overset{k_3}{\rightleftarrows}} C_3H_6O \cdot S + S + H^+ + e^- \quad (S3)$$

$$C_3H_6O \cdot S \underset{k_{-4}}{\overset{k_4}{\rightleftarrows}} C_3H_6O + S \quad (S4)$$

Rate calculations:

$$r_1 = k_1 c_{IPA}\theta_* - k_{-1}\theta_{IPA} \quad (S5)$$

$$r_2 = k_2\theta_{IPA}\theta_* - k_{-2}\theta_{C_3H_7O}\theta_* c_{H^+} \quad (S6)$$

$$r_3 = k_3\theta_{C_3H_7O}\theta_* - k_{-3}\theta_{ACE}\theta_* c_{H^+} \quad (S7)$$

$$r_4 = k_4\theta_{ACE} - k_{-4}c_{ACE}\theta_* \quad (S8)$$

1. If Step 2 is RDS and neglect the concentration of product (c_{ACE}) and coverage of ACE (θ_{ACE}):

$$\theta_{ACE} = c_{ACE} = 0 \quad (S9)$$

$$r_1 = k_1 c_{IPA}\theta_* - k_{-1}\theta_{IPA} = 0 \ \Rightarrow \ \theta_{IPA} = K_1 c_{IPA}\theta_* \quad (S10)$$

$$r_3 = k_3\theta_{C_3H_7O}\theta_* - k_{-3}\theta_{ACE}\theta_* c_{H^+} = 0 \Rightarrow \ \theta_{C_3H_7O} = 0 \quad (S11)$$

$$\theta_* + \theta_{IPA} = 1 \Rightarrow \theta_* = \frac{1}{1 + K_1 c_{IPA}} \quad (S12)$$

Substitute Equation 10, 11, 12 to Equation 6:

$$r = r_2 = \frac{K_1 k_2 c_{IPA}}{(1 + K_1 c_{IPA})^2} \quad (S13)$$

2. If Step 3 is RDS and neglect the concentration of product (c_{ACE}) and coverage of ACE (θ_{ACE}):

$$\theta_{ACE} = c_{ACE} = 0 \quad (S14)$$

$$r_1 = k_1 c_{IPA}\theta_* - k_{-1}\theta_{IPA} = 0 \ \Rightarrow \ \theta_{IPA} = K_1 c_{IPA}\theta_* \quad (S15)$$

$$r_2 = k_2\theta_{IPA}\theta_* - k_{-2}\theta_{C_3H_7O}\theta_* c_{H^+} = 0 \Rightarrow \ \theta_{C_3H_7O} = \frac{K_2\theta_{IPA}}{c_{H^+}} = \frac{K_1K_2c_{IPA}\theta_*}{c_{H^+}} \quad (S16)$$

$$\theta_* + \theta_{IPA} + \theta_{C_3H_7O} = 1 \Rightarrow \theta_* = \frac{1}{1 + K_1 c_{IPA} + \frac{K_1K_2c_{IPA}}{c_{H^+}}} \quad (S17)$$

Substitute Equation 16, 17 to Equation 7:

$$r = r_3 = \frac{K_1K_2k_3c_{IPA}}{c_{H^+}\left(1 + K_1c_{IPA} + \frac{K_1K_2c_{IPA}}{c_{H^+}}\right)^2} \quad (S18)$$

3. If Step 4 is RDS and neglect the concentration of product (c_{ACE}):

$$c_{ACE} = 0 \quad (S19)$$

$$r_1 = k_1 c_{IPA}\theta_* - k_{-1}\theta_{IPA} = 0 \ \Rightarrow \ \theta_{IPA} = K_1 c_{IPA}\theta_* \quad (S20)$$

$$r_2 = k_2\theta_{IPA}\theta_* - k_{-2}\theta_{C_3H_7O}\theta_* c_{H^+} = 0 \Rightarrow \theta_{C_3H_7O} = \frac{K_2\theta_{IPA}}{c_{H^+}} = \frac{K_1K_2c_{IPA}\theta_*}{c_{H^+}} \quad (S21)$$

$$r_3 = k_3\theta_{C_3H_7O}\theta_* - k_{-3}\theta_{ACE}\theta_* c_{H^+} = 0 \Rightarrow \theta_{ACE} = \frac{K_3\theta_{C_3H_7O}}{c_{H^+}} = \frac{K_1K_2K_3c_{IPA}\theta_*}{{c_{H^+}}^2} \quad (S22)$$

$$\theta_* + \theta_{IPA} + \theta_{C_3H_7O} + \theta_{ACE} = 1 \Rightarrow \theta_* = \frac{1}{1 + K_1c_{IPA} + \frac{K_1K_2c_{IPA}}{c_{H^+}} + \frac{K_1K_2K_3c_{IPA}}{{c_{H^+}}^2}} \quad (S23)$$

Substitute Equation 19-23 to Equation 8:

$$r = r_4 = \frac{K_1K_2K_3c_{IPA}\theta_*}{{c_{H^+}}^2} = \frac{K_1K_2K_3c_{IPA}}{{c_{H^+}}^2 + K_1c_{IPA}{c_{H^+}}^2 + K_1K_2c_{IPA}c_{H^+} + K_1K_2K_3c_{IPA}} \quad (S24)$$

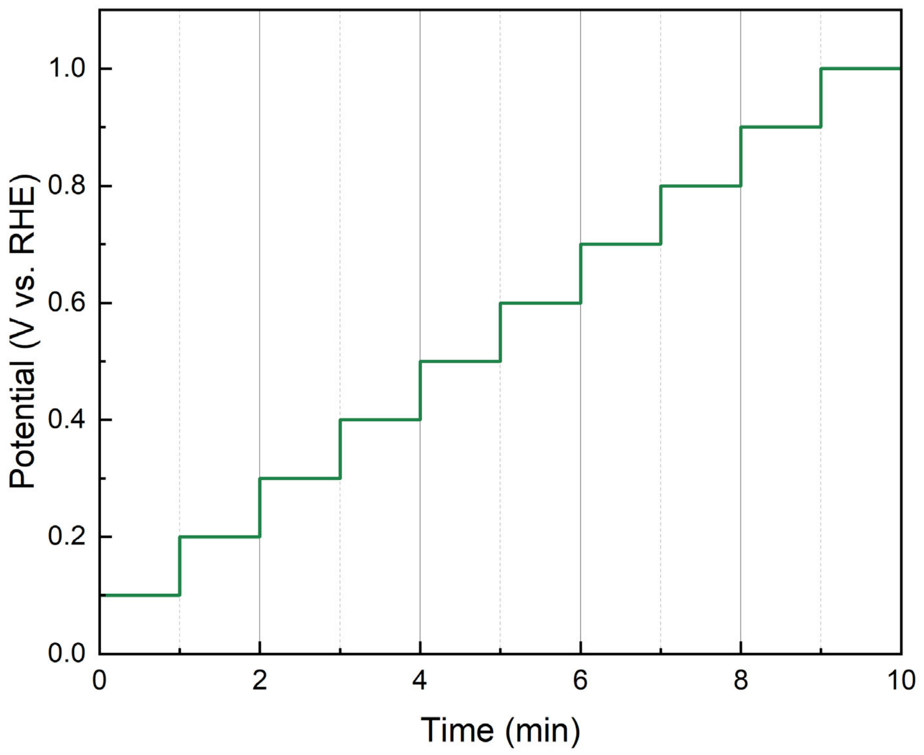

Figure S20. Plot of potential to time for in-situ AIR-FTIR from 0 – 1.0 V vs. RHE.

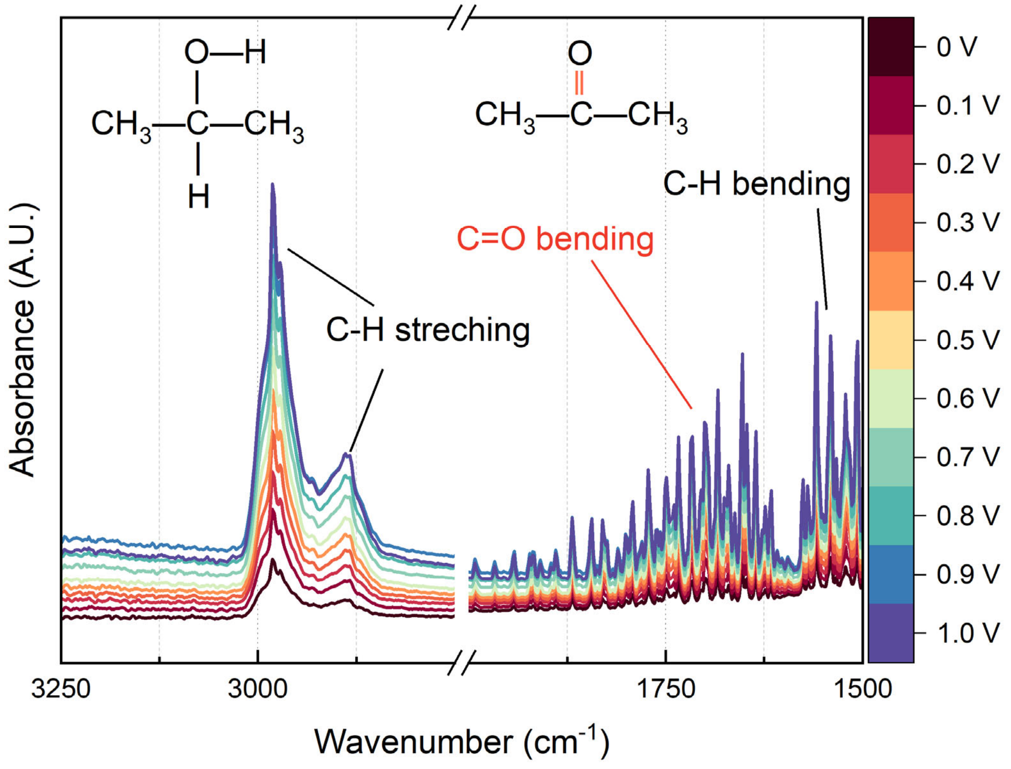

Figure S21. In-situ AIR-FTIR spectra of IPA oxidation on Pt catalyst from 0 – 1.0 V vs. RHE.

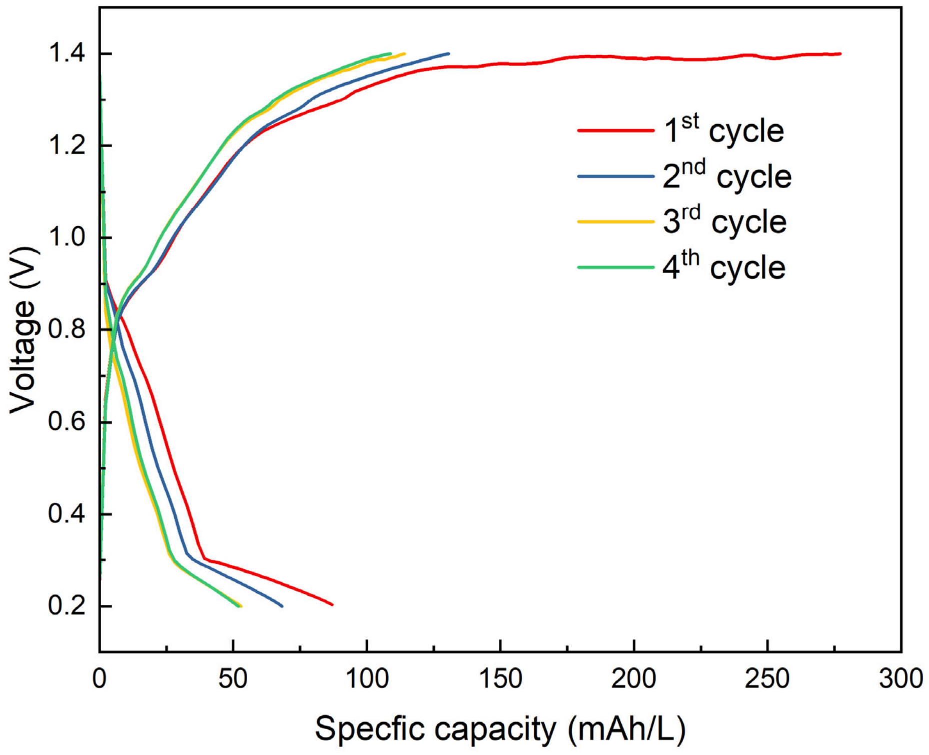

Figure S22. Galvanostatic charge-discharge profiles of IPA/ACE-V(IV)/V(V) H-cell.
